# Physiological effects of invasive ventilation with neurally adjusted ventilatory assist (NAVA) in a crossover study

**DOI:** 10.1186/s12887-016-0717-4

**Published:** 2016-11-08

**Authors:** Jean-Michel Liet, François Barrière, Bénédicte Gaillard-Le Roux, Pierre Bourgoin, Arnaud Legrand, Nicolas Joram

**Affiliations:** 1 Pediatric Intensive Care Unit, Hôpital Femme-Enfant-Adolescent, the University Hospital Center of Nantes (CHU), 38 bd Jean-Monnet, 44093 Nantes, France; 2 CIC-INSERM 1413, University of Nantes, Nantes, France

**Keywords:** Mechanical ventilation, Cardiac index, Doppler ultrasonography, Neurally adjusted ventilatory assist, Interactive ventilatory support, Electrical activity of diaphragm, Intensive care, Children, Infants, Cardiac surgery

## Abstract

**Background:**

Neurally Adjusted Ventilatory Assist (NAVA) is a mode of assisted mechanical ventilation that delivers inspiratory pressure proportionally to the electrical activity of the diaphragm. To date, no pediatric study has focused on the effects of NAVA on hemodynamic parameters. This physiologic study with a randomized cross-over design compared hemodynamic parameters when NAVA or conventional ventilation (CV) was applied.

**Methods:**

After a baseline period, infants received NAVA and CV in a randomized order during two consecutive 30-min periods. During the last 10 min of each period, respiratory and hemodynamic parameters were collected. No changes in PEEP, FiO_2_, sedation or inotropic doses were allowed during these two periods. The challenge was to keep minute volumes constant, with no changes in blood CO_2_ levels and in pH that may affect the results.

**Results:**

Six infants who had undergone cardiac surgery (mean age 7.8 ± 4.1 months) were studied after parental consent. Four of them had low central venous oxygen saturation (ScvO_2_ < 65 %). The ventilatory settings resulted in similar minute volumes (1.7 ± 0.4 vs. 1.6 ± 0.6 ml/kg, *P* = 0.67) and in similar tidal volumes respectively with NAVA and with CV. There were no statistically significant differences on blood pH levels between the two modes of ventilation (7.32 ± 0.02 vs. 7.32 ± 0.04, *P* = 0.34). Ventilation with NAVA delivered lower peak inspiratory pressures than with CV: -32.7 % (95 % CI: -48.2 to –17.1 %, *P* = 0.04). With regard to hemodynamics, systolic arterial pressures were higher using NAVA: +8.4 % (95 % CI: +3.3 to +13.6 %, *P* = 0.03). There were no statistically significant differences on cardiac index between the two modes of ventilation. However, all children with a low baseline ScvO_2_ (<65 %) tended to increase their cardiac index with NAVA compared to CV: 2.03 ± 0.30 vs. 1.91 ± 0.39 L/min.m^2^ (median ± interquartile, *P* = 0.07).

**Conclusions:**

This pilot study raises the hypothesis that NAVA could have beneficial effects on hemodynamics in children when compared to a conventional ventilatory mode that delivered identical PEEP and similar minute volumes.

**Trial registration:**

ClinicalTrials.gov Identifier: NCT01490710. Date of registration: December 7, 2011.

## Background

To better match the level of ventilator assistance to the patient’s needs, manufacturers have developed new modes that deliver a level of assistance proportional either to the patient’s inspiratory muscle effort, with proportional assist ventilation (PAV); or to the diaphragmatic electrical activity (EAdi), with neurally adjusted ventilatory assist (NAVA) [1]. For now, only NAVA is usable for infants. With this mode of mechanical ventilation, the collected electrical signal allows synchronization of ventilation to spontaneous breathing efforts of the child, as well as permitting pressure assistance proportional to the electrical signal. NAVA provides both fine synchronization of respiratory support and pressure assistance varying with the needs of the child. To our knowledge, no data have been published on the impact of NAVA on the hemodynamics in children mechanically ventilated.

The impact of mechanical ventilation on the circulatory system remains a concern in some infants following surgery for repair of congenital cardiac defects [2]. After the completion of the operation, when there is consensus regarding a good surgical result, many children nowadays are rapidly weaned and successfully separated from mechanical ventilation shortly after the procedure. However, duration of mechanical ventilation is inversely proportional to the patient’s age and directly related to the duration of cardiopulmonary bypass and complexity of the surgical procedure [3].

This physiologic study with a randomized cross-over design compared hemodynamic parameters when neurally adjusted ventilatory assist (NAVA) or conventional ventilation (CV) was applied.

## Methods

This study was performed in the 12-bed PICU of the university hospital center of Nantes (France), between June 2012 and March 2013. Last year, 712 children were admitted in this unit. Among them, 182 underwent a cardiac surgery with cardiopulmonary bypass (55 % were extubated within 8 h). This trial was registered (ClinicalTrials.gov Identifier: NCT01490710). Research has been performed in accordance with the Declaration of Helsinki and has been approved by an appropriate ethics committee (*Comité de Protection des Personnes Ouest III de POITIERS, Réference comité: Protocole n°11.07.20*). The parents of all the children gave their written consent.

### Study design: prospective randomized cross-over study

**Fig. 1:**
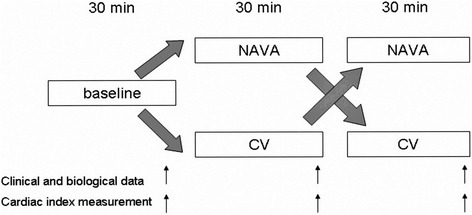
Study design. Baseline measures were collected when patient met stability conditions. No changes in PEEP, FiO_2_, sedation nor inotropic doses were allowed during the two 30-min study periods. NAVA = Neurally Adjusted Ventilatory Assist; CV = Conventional Ventilation

After a baseline period, children received conventional ventilation (CV) and NAVA during two consecutive periods of 30 min, in random order. This randomization was performed by contacting a server to define the order in which the two modes of ventilation were administered. There was no washout time between these two periods. During the last 10 min of each period at a steady state, hemodynamic parameters, respiratory parameters, blood gas and cardiac index were collected. No changes in PEEP, FiO_2_, sedation or inotropic doses were allowed during these two periods. The study protocol is depicted in Fig. 1.

### Participants

Children hospitalized for postoperative care after cardiac surgery were considered for inclusion. Inclusion criteria were: admission weight >5 kg; invasive ventilatory support ongoing; NAVA ventilation available; sedation status allowing the NAVA ventilation functioning. Non inclusion criteria were: parental refusal; oesophageal disease prohibiting gastric tube; brain damage conflicting with spontaneous ventilation; clinical instability requiring treatment and/or management inconsistent with research.

### Intervention

Children were ventilated with a Servo-i ventilator (Maquet Critical Care, Solna, Sweden). During a baseline period, conventional ventilation was applied and adjusted according to blood gases. After the baseline period in which NAVA probe and Doppler monitoring were introduced, all children received conventional ventilation (CV) and NAVA during two consecutive periods of 30 min, in random order. The ventilation settings were supposed to provide the same minute volumes.

#### Conventional ventilation (CV)

With the goal of providing the lowest peak pressures, we used the Volume Control Intermittent Mandatory Ventilation (VC-IMV). Ventilatory parameters were as follows: tidal volume (between 5 and 8 ml/kg), respiratory rate (between 20 and 30 cycles/min). Pressure Support was set at 12 cmH_2_O above the PEEP. A flow pneumatic trigger was used to synchronize the support to spontaneous breaths. The flow pattern during the volume control ventilation was constant flow. The inspiratory time was initially set at 0.5 s and adjusted according to the inspiratory flow waveforms if needed. The respiratory rate of VC-IMV was set at a high level (between 20 and 30 cycles/min), not allowing many spontaneous cycles. The CV settings were adjusted during the baseline period with no changes allowed during the study period.

#### Neurally Adjusted Ventilatory Assist (NAVA)

During NAVA, the ventilator delivers a pressure proportional to the electrical activity of the diaphragm (EAdi). The settings in NAVA mode included PEEP, FiO_2_ and level of NAVA assistance. The NAVA level was set to 1 cmH_2_O/μV. We had previously observed that children ventilated with NAVA usually adjust their EAdi to maintain a normal blood pH by preserving physiologic tidal volumes and minute volumes. This is no longer true when children are highly sedated. Backup ventilation was Pressure Control set at 12 cmH_2_O above PEEP in case of failure EAdi signal detection. During NAVA, the positive pressure is triggered when the deflection in the EAdi curve exceeds 0.5 μV, and is cycled-off when the EAdi drops to 70 % of its peak value.

### Outcomes measurements

#### Respiratory measurements

Peak inspiratory pressures (PIP), PEEP, FiO_2_, Tidal volume (V_T_), Minute volume, EADI max, Respiratory rate, were recorded for all infants during the last 10 min of each period. Measurements of each parameter were repeated at steady state a total of six times to guarantee reliability and reproducibility. We collected the most representative values rather than gathering data at constant interval because of the variability of the respiratory measurements in NAVA. Hemodynamic and respiratory parameters were collected always in the same time. Respiratory system compliance (Crs) according to the ventilation mode was estimated by the ratio V_T_/(PIP-PEEP) in ml/kg.cmH_2_O.

#### Hemodynamic measurements

Cardiac index (CI, L/min.m^2^) was assessed by transesophageal Doppler ultrasonography (CardioQP, Gamida, France) [4]. Other hemodynamic parameters included heart rate, arterial pressures (by means of an arterial catheter placed before the surgery), and central venous pressure (by means of a central venous catheter inserted into the right internal jugular vein). Cerebral Near InfraRed Spectroscopy (NIRS) was measured with a non invasive optical monitor of regional cerebral oxygen saturation (Invos Oximeter, Covidien, Boulder, CO). The pulse oximeter perfusion index was provided by the monitors (Intellivue MP70 monitor, Philips Medical Systems) [5]. Hemodynamic parameters were collected always in the same time than respiratory parameters. Left cardiac work index [6] was calculated as: LCWI (kg.min/m^2^) = CI × MAP × 0.0136, with CI for cardiac index and MAP for mean arterial pressure.

#### Biological measurements

At the end of each period (CV and NAVA), a blood sample was taken through the internal jugular catheter to measure pH, PCO_2_, and central venous oxygen saturation (ScvO_2_). These measurements were performed in the PICU by a blood gases analyser (GEM Premiere 4000, Instrumentation Laboratory UK Ltd).

### Statistical analysis

Each parameter collected in the last 10 min of each study period was averaged, and compared by a Wilcoxon test for paired samples. Measurements in NAVA and CV are reported as median (interquartile). Changes in parameters during NAVA compared to CV are expressed as mean percentage (95 % CI, *P*). The *P*-value taken to indicate significance was *P* < 0.05. For this pilot study no sample size was calculated.

## Results

The study was concluded after one year. Technical problems were greater than expected: a measure of cardiac output sufficiently accurate, and especially stable in time, was very difficult to reach in children who already had a stomach tube for the NAVA ventilation. We asked the research office of our institution for continuing recruiting more patients. In view of the technical difficulties we had, this request was not accepted.

Nine children were randomized. The quality of the surgical repair was always checked by a cardiologist prior to inclusion in the study. Analysis was performed on only six of them. We were unable to start NAVA on one child due to a lack of EAdi signal capture. Thereafter, when the NAVA signal was not stable enough, children were not included. In two other children we were unable to record a Doppler signal likely due to interference between the CardioQP probe and the EAdi probe both intra oesophageal placed. Patient characteristics are provided in Table 1. The mean (± SD) age was 7.8 ± 4.1 months, and weight was 6.7 ± 1.2 kg. Four children had low central venous saturation (ScvO_2_ < 65 %) at the baseline period: the children 1, 2, 3, 6 in the Table 1.

**Table 1:** Children characteristics

Child	1	2	3	4	5	6
Gender	M	M	M	F	F	F
Age (mo)	3	13	4	10	6	11
Weight (kg)	5.7	8.9	7.0	6.0	5.8	7.0
Cardiopathy	AVSD	VSD with pulmonary stenosis	tetralogy of Fallot	atrial septal defect	coarctation of the aorta	pulmonary atresia with VSD
Cardiopulmonary bypass duration (min)	96	83	130	48	0	130
Time between PICU entry and study (hours)	20	7	20	4	5	7
Baseline cardiac index (L/min.m^2^)	2.00	2.85	1.93	2.60	2.75	1.85
Milrinone (mcg/kg/min)	0.9	0.5	0.3	0.5	0	0.5
Adrenaline (mcg/kg/min)	0	0	0	0	0	0.05
ScvO_2_ (%)	64	63	52	82	68	52
Perfusion index (%)	0.31	3.65	0.60	3.62	1.90	0.87
Random order	CV then NAVA	NAVA then CV	CV then NAVA	CV then NAVA	NAVA then CV	NAVA then CV

All children were receiving morphine 0.5 mg per kilo per day. All, except the 2 first, were receiving midazolam 40 micrograms per kilo per hour

*AVSD* atrioventricular septal defect, *VSD* ventricular septal defect, *NAVA* neurally adjusted ventilatory assist, *CV* conventional ventilation, *ScvO*_*2*:_ central venous oxygen saturation

Respiratory parameters, hemodynamic parameters, and blood gases are provided in Table 2. The ventilatory settings resulted in similar minute volumes and in similar tidal volumes in NAVA compared to CV. These similar minute volumes in each periods of the study were supposed when the study was designed. Nevertheless, significant lower peak inspiratory pressures (PIP) were observed during NAVA compared to CV. Apparent respiratory system compliance improved with NAVA in all children. We observed higher systolic arterial pressures during NAVA compared to CV. Pulse oximeter perfusion index were also higher during NAVA.

**Table 2:** Respiratory parameters, hemodynamics and biological data

Respiratory parameters	CV	NAVA	NAVA versus CV	*P*
Respiratory rate (/min)	30 (7)	35 (13)	+26.4 (-3.6, +56.4)	0.17
V_T_ (ml/kg)	6.6 (0.7)	6.9 (0.3)	+5.3 (-5.5, +16.1)	0.46
Minute volume (L/min)	1.7 (0.4)	1.6 (0.6)	+5.4 (-4.3, +15.1)	0.67
PIP (cm H_2_O)	21 (6)	11 (4)	-32.7 (-48.2, -17.1)	0.04
PEEP (cm H_2_O)	4 (2)	4 (2)	-	(a)
EAdi max (microVolt)	3.9 (3.8)	6.3 (1.4)	+61.2 (-4.6, +126.9)	0.34
SpO_2_ (%)	98 (3)	96 (3)	-1.2 (-2.8, +0.5)	0.14
FiO_2_	30 (4)	30 (4)	-	(a)
Crs (ml/kg.cm H_2_O)	0.37 (0.19)	0.87 (0.32)	+98.4 (+43.8, +153.0)	0.04
Hemodynamic parameters				
Heart rate	156 (15)	156 (22)	+2.1 (-0.7, +4.5)	0.17
Systolic arterial pressure (mmHg)	93 (6)	99 (13)	+8.4 (+3.3, +13.6)	0.03
Diastolic arterial pressure (mmHg)	54 (12)	57 (6)	+3.6 (-3.0, +10.1)	0.46
Central venous pressure (mmHg)	11 (5)	10 (5)	+3.9 (-5.3, +13.1)	0.92
Cerebral NIRS (%)	62 (5)	61 (3)	+1.6 (-2.6, +5.8)	0.34
Cardiac index (L/min.m^2^)	2.33 (0.84)	2.26 (0.70)	+1.4 (-3.4, +6.2)	0.17
Pulse oximeter perfusion index (%)	1.50 (2.45)	1.78 (2.29)	+18.8 (+3.0, +34.7)	0.04
Venous blood gases				
pH	7.32 (0.04)	7.32 (0.02)	-0.1 (-0.4, +0.2)	0.34
PCO_2_ (mm Hg)	47.3 (5.1)	45.8 (8.1)	+0.6 (-5.1, +6.3)	0.50
ScvO_2_ (%)	60.1 (20.9)	58.4 (15.4)	+3.3 (-5.7, +12.2)	0.60

Measurements in NAVA and CV are reported as median (interquartile). Variations between NAVA versus CV are reported as mean percentage (95 % CI). Statistical analyses between NAVA versus CV were performed by a Wilcoxon test for paired samples

(a) No changes in PEEP, FiO_2_, neither in sedation or inotropic doses were allowed during these study periods

*NAVA* Neurally adjusted Ventilatory Assist, *CV* Conventional Ventilation, *V*_*T*_ tidal volume, *PIP* peak inspiratory pressure, *Crs* respiratory system compliance, *EAdi* electrical activity of the diaphragm, *ScvO*_*2*_ central venous oxygen saturation

**Fig. 2:**
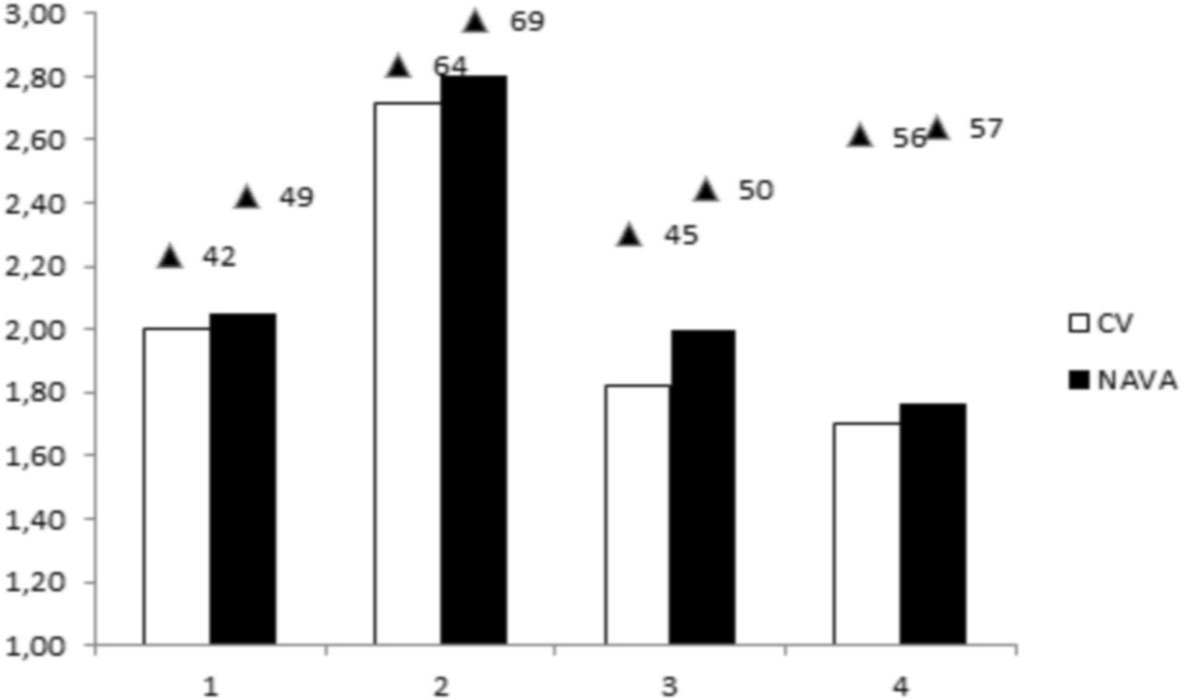
Cardiac index and ScvO_2_ of the low baseline ScvO_2_ infants, according to the ventilation mode. *White bars* are cardiac index when CV is applied; *back bars* are cardiac index when NAVA is applied. The *triangles* above the bars indicate the values of the corresponding ScvO_2_ (%). ScvO_2_ = central venous oxygen saturation; NAVA = Neurally adjusted Ventilatory Assist; CV = Conventional Ventilation

Cardiac index during NAVA compared to CV did not statistically differ. However, all four children with ScvO_2_ < 65 % tended to have higher cardiac index after 30 min of ventilation with NAVA compared to CV: 2.03 ± 0.30 vs. 1.91 ± 0.39 L/min.m^2^ (median ± interquartile, *P* = 0.07). Individual values of cardiac index and ScvO_2_ of children with low baseline ScvO_2_ are shown in Fig. 2. Upper values of pulse oximeter perfusion index were also observed. Turning now to left cardiac work index, the increase is +12.4 % (95 % CI: +3.8 % to +20.9 %, *P* = 0.07).

## Discussion

NAVA can be used in infants receiving postoperative mechanical ventilation after congenital heart surgery, as previously reported [7–10]. Infants included in this study all weighed less than 10 kg and had been operated less than 24 h before. To deliver a same tidal volume, NAVA required lower inspiratory pressures than conventional ventilation and had some beneficial effects on hemodynamics. Several data validate that the children were not over assisted when ventilated in CV: the delivered volumes, EAdi max, PCO_2_ and pH are similar in CV compared to NAVA. We suggest that lower EAdi peaks observed in CV are related to the normalization of the blood pH by CV ventilation. If children were over-assisted in CV, EAdi would have been missing, and minute volume would have been high, with a blood alkalosis.

In a large randomized controlled trial where NAVA was used as a primary mode of ventilation, lower peak inspiratory pressures were found in the NAVA group [11]. It looks as if gas volume delivery, and probably alveolar expansion, required less pressure. This could be enhanced by an improvement in patient–ventilator interactions with a greater respiratory variability as reported in previous studies [12–16].

The effectiveness of this mode of mechanical ventilation was associated with some beneficial effects on the hemodynamics. Systolic arterial pressures and pulse oximeter perfusion index were significantly higher in NAVA. This pulse oximeter perfusion index provides a monitoring of illness severity in neonates [5]. In fact, we observed that the children with a low cardiac index had also a low perfusion index. This index was significantly higher when the children were ventilated with NAVA compared to CV.

The overall values of the cardiac index and of the central venous oxygen saturation during NAVA compared to CV were not significantly higher, but there are possibly no reasons to observe an increase in these two parameters when tissue perfusion is already effective. And actually, the four children with a low ScvO_2_ trend to have a higher cardiac index after 30 min of ventilation with NAVA (Fig. 2). Moreover, this increase in cardiac index is associated with higher systemic pressures and then higher left cardiac work index. Lower oxygen content in venous blood usually reflects an oxygen balance disrupted between oxygen supply and demand. Ventilation with NAVA could allow the body to increase the cardiac output to ensure adequate oxygen availability. These children had congenital heart disease affecting the right heart: VSD with pulmonary stenosis, tetralogy of Fallot, and pulmonary atresia with VSD. Positive pressure ventilation and PEEP often result in increased right ventricular afterload due to capillary compression [3, 17]. Lower intrathoracic pressures in NAVA than in conventional ventilation could improve the right ventricular function during inspiration by reducing the right ventricular afterload.

An improvement in apparent respiratory system compliance was observed when NAVA is applied. Cyclic intrathoracic pressure changes, characteristic of spontaneous breathing, could be preserved. Both fine synchronization of respiratory support and pressure assistance varying with the needs and the spontaneous breathing of the child could improve the pulmonary compliance. Nonetheless, it is important to note that we did not record the transpulmonary pressure. It is likely that, while the airway pressure decreased during NAVA, an increase in esophageal pressure swings occurred because of the patient respiratory efforts. This could have participated in the decrease in apparent compliance improvement [18].

This study has some limitations. Firstly, few children were studied. Many children dropped out due to early extubation in the operation room or immediately on arrival in the intensive care unit. Technical problems were greater than expected: a measure of cardiac output sufficiently accurate, and especially stable in time, was very difficult to reach in children who already had a stomach tube for the NAVA ventilation. Nevertheless the cross-over design of this study has two advantages over both a parallel study and a non-crossover longitudinal study [19]. The influence of confounding covariates is reduced because each cross-over patient serves as his or her own control. Cross-over designs are also statistically efficient and require fewer subjects than do non-crossover designs (even other repeated measures designs). Secondly, the agreement of the cardiac output measurement between the transoesophageal Doppler probe using CardioQP and the thermodilution technique during heart catheterisation in paediatric patients was described as weak [20]. However the CardioQP seems to be capable of detecting slight changes in cardiac output for critically ill children [21, 22]. This bed-side device for cardiac output measurement is minimally invasive, and provides better results in monitoring the slight variations rather than in measuring absolute values. As we were performing a cross-over study, we were interested primarily by changes. Finally, only comparison with CV-SIMV with relatively high preset respiratory rate preventing many spontaneous cycles was made, not with other ventilation strategies.

## Conclusion

In conclusion, this cross-over study provides new data on the NAVA ventilation. This pilot study raises the hypothesis that a ventilatory assistance with NAVA could provide improvements in hemodynamics when compared to a conventional ventilatory mode that delivered identical PEEP and similar minute volumes. Thus, because minute volumes were not different between the two modes of ventilation, hemodynamic effects are linked to NAVA mode itself and NOT to incorrect settings in conventional ventilation. Further studies with larger population are needed to confirm these promising results.

## Abbreviations

AVSD: Atrioventricular septal defect
CI: Cardiac index
Crs: Respiratory system compliance
CV: Conventional ventilation
EAdi: Diaphragmatic electrical activity
LCWI: Left cardiac work index
NAVA: Neurally adjusted ventilatory assist
NIRS: Cerebral near infrared spectroscopy
PAV: Proportional assist ventilation
PIP: Peak inspiratory pressures
ScvO_2_: Central venous oxygen saturation
VC-IMV: Volume control intermittent mandatory ventilation
VSD: Ventricular septal defect
VT: Tidal volume
